# Ethics is the best professional policy^☆^

**DOI:** 10.1016/j.bjorl.2016.09.007

**Published:** 2016-10-27

**Authors:** Aracy Pereira Silveira Balbani

**Affiliations:** Private practice, Tatuí, SP, Brazil

Dear Editor,

The editorial “The Future … to whom it belongs?”^1^ raises two considerations on our professional practice.

The first one, stimulated by our colleague Marcio Abrahão, refers to the Brazilian tax burden. The National Union of Attorneys of the National Treasury estimates that the cost of the crime of tax evasion in Brazil in 2015 alone was more than R$ 420 billion – seven times more than the annual cost of corruption. Therefore, there is no way to attain significant relief to the pockets of honest taxpayers without tax reform, repression of tax evasion and punishment of tax evaders.^2^ If individuals and businesses do not evade taxes, it will be possible to reduce tax rates and the tax burden will weigh less on each of us. Therefore, we endorse the editorial on the role of medical institutions in the national political scenario, because society's revulsion against crime gets a stronger voice through prestigious organizations, such as the ABORL-CCF. There is nothing more appropriate for our Association to do than to start or join a public campaign to combat tax evasion.

The second consideration concerns the ethical and humanitarian nature of Medicine. It is incontestable that we need to demand a fair and decent remuneration for all workers, including doctors. In parallel, the current crises in economic and moral values bring us doctors another important mission: volunteering for the benefit of those who suffer the most. Our defenseless countrymen rely on us for more than solving their health problems. They also ask for our brave actions as citizens, so that together, we can demand good public health policies and reject hatred, prejudice, injustice, social inequality, threats to peace and disregard for human life.

Respecting the secular state, we must have the serene consciousness to honor every day the passage from the “Physician's Prayer”, by Maimonides (1135–1204): “(…) to serve the rich and the poor, the good and the wicked, friends and enemies, and may I never see in the patient anything but a fellow creature in pain.” Ethics is the noble policy that can lead us to a better future.

## Conflicts of interest

The author declares no conflicts of interest.
